# A Cre-lox approach for transient transgene expression in neural precursor cells and long-term tracking of their progeny *in vitro *and *in vivo*

**DOI:** 10.1186/1471-213X-7-45

**Published:** 2007-05-15

**Authors:** Cédric G Geoffroy, Olivier Raineteau

**Affiliations:** 1 Department of Clinical Neurosciences, Cambridge centre for Brain Repair, University of Cambridge, Robinson Way, Cambridge CB2 2PY, UK

## Abstract

**Background:**

Neural precursor cells (NPCs) can be isolated from various regions of the postnatal central nervous system (CNS). Manipulation of gene expression in these cells offers a promising strategy to manipulate their fate both *in vitro *and *in vivo*. In this study, we developed a technique that allows the transient manipulation of single/multiple gene expression in NPCs *in vitro*, and the long-term tracking of their progeny both *in vitro *and *in vivo*.

**Results:**

In order to combine the advantages of transient transfection with the long-term tracking of the transfected cells progeny, we developed a new approach based on the cre-lox technology. We first established a fast and reliable protocol to isolate and culture NPCs as monolayer, from the spinal cord of neonatal transgenic Rosa26-YFP cre-reporter mice. These cells could be reliably transfected with single/multiple plasmids by nucleofection. Nucleofection with mono- or bicistronic plasmids containing the Cre recombinase gene resulted in efficient recombination and the long-term expression of the YFP-reporter gene. The transient cre-expression was non-toxic for the transfected cells and did not alter their intrinsic properties. Finally, we demonstrated that cre-transfected cells could be transplanted into the adult brain, where they maintained YFP expression permitting long-term tracking of their migration and differentiation.

**Conclusion:**

This approach allows single/multiple genes to be manipulated in NPCs, while at the same time allowing long-term tracking of the transfected cells progeny to be analyzed both *in vitro *and *in vivo*.

## Background

It is now well documented that neural precursor cells (NPCs) are present in both neurogenic (i.e. dentate gyrus, DG, and subventricular zone, SVZ)[1] and non neurogenic (e.g. spinal cord)[2,3] regions of the postnatal central nervous system (CNS). Such cells have been successfully isolated *in vitro *from many species, including rodents[4], and from tissues of different developmental ages[5]. *In vitro*, NPCs proliferate for extensive periods of time and can differentiate into the three cell types of the CNS (i.e. oligodendrocytes, astrocytes and neurons) following the withdrawal of mitotic factors. These cells may therefore represent a source of transplantable material for replacement therapies after CNS injury or in neurodegenerative disease[6,7].

Following transplantation, the fate of NPCs appears to be highly dependent on their site of engraftment. Thus, NPCs isolated from various CNS regions are able to give rise to neurons when transplanted into the subventricular zone or dentate gyrus subgranular zone[3]. In contrast, the same cells invariably adopt a glial fate when transplanted outside these discrete neurogenic regions[8,9]. A potential strategy to overcome the gliogenic environment of most postnatal CNS regions is the manipulation of NPCs gene expression before transplantation in order to promote a desired fate. Genes of particular interest include those for transcription factors which are involved in the determination and differentiation of multiple neural lineages during development[10]. Recent reports indicate that transcription factors over-expression in isolated NPCs *in vitro*, can direct their differentiation toward a given cell fate [11-14]. Furthermore, transplantation experiments demonstrate the potential of this strategy in overcoming the gliogenic signals of the adult CNS[13], albeit to a lesser extent.

In order to explore this strategy, new techniques that allow rapid assessment of the neurogenic properties of different proneural transcription factors have to be developed. Because long-term expression of transcription factors is non-physiological and may be harmful to the transfected cells[15], transient expression should be favoured. Moreover, the gliogenic/neurogenic molecular pathways are complex and highly intermingled [16-18]. It is therefore likely that the concomitant expression of multiple genes will need to be achieved in order to obtain a strong and environment-insensitive neurogenic effect. Furthermore, despite transient transcription factor expression, long-term labelling of the transfected cell population must be obtained in order to assess the fate of the manipulated cells.

In this study, we used a transient Cre-loxP system to induce stable YFP expression in NPCs isolated from the spinal cord of neonatal transgenic cre-reporter mice. These cells were cultured as a monolayer and transfected by nucleofection with mono- or bicistronic plasmids containing the gene coding for Cre recombinase. We showed that such transfection was transient, reliable and was able to mediate a stable expression of the YFP reporter gene, both *in vitro *and *in vivo*. This system did not alter the intrinsic properties of the cells and permitted a high rate of cotransfection. This approach will be valuable for the rapid screening of transcriptions factor over expression in NPCs, and the long-term tracking of their progeny *in vitro *and *in vivo*.

## Results

### Isolation of neural precursors cells from Cre-reporter mice

Neurosphere cultures still represent the method of choice for rapid and reliable isolation of neural precursor cells (NPCs) from the central nervous system. However, because of their heterogeneity and lack of accessibility (e.g. for transfection experiments or cell fate analysis), alternative culture techniques allowing the growth of NPCs as monolayers have now emerged to become the technique of choice in many laboratories.

**Figure 1:**
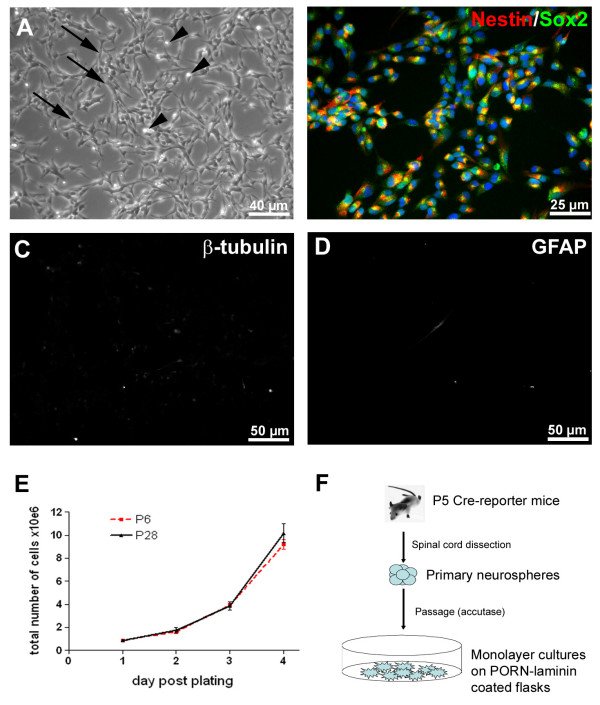
**Isolation and maintenance of neural precursors cells (NPCs) from cre-reporter mice postnatal spinal cord as monolayer cultures**. **A**: Image of live NPCs from mouse spinal cord monolayer cultures. Arrows show cells with large, flat and dark cell body with multiple processes. Arrow heads show fast proliferating cells with a small, spherical and bright cell body. **B-D**: Immunostaining analysis of NPC monolayer cultures. In the presence of mitotic factors (FGF-2 20 ng/ml & EGF 20 ng/ml), virtually all the cells co-expressed Nestin and Sox2 (red and green, respectively **B**) and none express either βIII-tubulin (**C**) or GFAP (**D**). **E**: Growth curves of spinal cord NPCs showed an identical exponential expansion at passage 6 and 28, with a population doubling time of ~24 h. **F**: Schematic protocol for the culture of NPCs from P5 mouse spinal cord as monolayers on polyornithine-laminin substrate.

Following initial isolation as neurospheres, dissociated NPCs from the postnatal spinal cord were plated onto poly-ornithine/laminin coated flasks. Cells attached to the coated plastic and were thereafter successfully expanded using a monolayer culture approach (Fig. 1A and 1E). Some cells presented a large, flat and dark cell body with some processes (arrow, Fig. 1A), while others appeared more spherical, bright and without extensions (arrowhead, Fig. 1A). This second cell population divided intensely as revealed by time-lapse imaging (data not shown). In the presence of the mitogenic factors EGF and FGF2 (20 ng/ml), monolayer cultures proliferated robustly, with a population doubling time of ~24 h. (Fig. 1E). Given that progenitor cells do not exhibit long-term self-renewal, and only cultures containing stem cells may be expanded continuously, we compared the proliferative capabilities of early and late passage monolayer cultures (i.e. passage 6 and passage 28, respectively, Fig. 1E). If the cultures did not contain a stem cell population their growth would be expected to fail over time. Cells from both passages presented a comparable exponential growth speed with no sign of a decrease in their proliferative capabilities.

**Figure 2:**
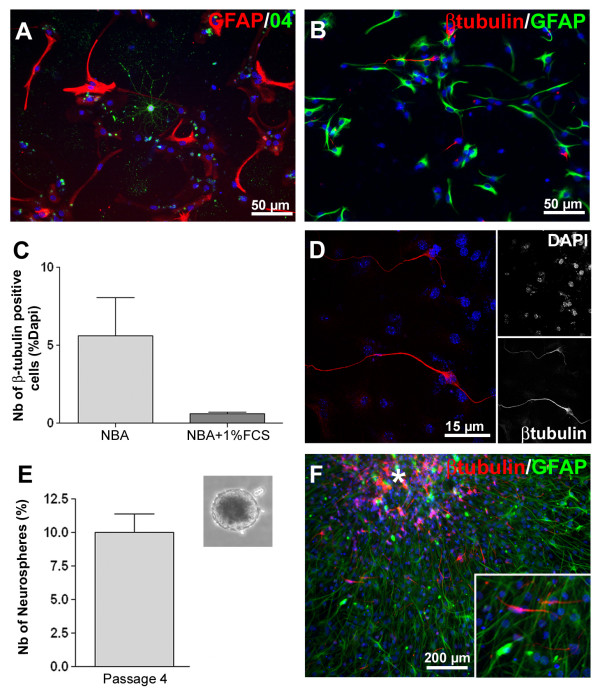
**Multipotency of postnatal mouse spinal cord NPCs grown as monolayers**. **A-D**: Upon removal of growth factors and in the presence of FCS (1%), cells quickly differentiated into oligodendrocytes (**A**, O4, green), neurons (βIII-tubulin, red, **B **&**D**), and astrocytes (GFAP, red in **A **and green in **B**). **C**: Monolayer NPCs respond differently to change in differentiation conditions. Removal of FCS from the differentiation medium significantly increased the number of βIII-tubulin-positive cells from 0,59% ± 0,40 to 5,7% ± 3,2 at 48 hours *in vitro*. **D**: Confocal picture of βIII-tubulin positive cells showing long neurites after 7 days in differentiating medium. **E-F**: Neurosphere formation by monolayer NPCs cultures. **E**: Percentage of neurospheres formed after 2 weeks in clonal density NPC cultures (passage 4). Insert shows a typical example of the neurospheres formed from early passage (P4) monolayer NPCs cultures. **F**: Neurospheres generated by clonal density cultures of monolayer NPCs, differentiated into astrocytes (GFAP, green) and neurons (βIII-tubulin, red). The asterisk indicates the location of the neurosphere.

In addition, immunostaining was performed in order to characterize undifferentiated and differentiated monolayer cultures. In the presence of the mitotic factors EGF and FGF-2, virtually all cells were positive for markers of undifferentiated neural cells, Nestin and Sox2 (Fig. 1B). Importantly, none of the cells expressed neither neuronal (βIII-tubulin, Fig. 1C) nor glial markers (GFAP for astrocytes Fig. 1D and O4 for precursors of oligodendrocytes, not shown) at this stage. Following the removal of the mitogenic factors and the addition of 1% FCS, the cells rapidly adopted a more complex radial or polarized morphology. Immunostaining for neuronal (i.e. βIII-tubulin) or glial (i.e. GFAP & O4) markers confirmed their differentiation into the three cell types of the central nervous system: neurons (0,59% ± 0,40%, Fig. 2B and 2D), astrocytes (37% ± 4,2% Fig. 2A and 2B) and oligodendrocytes (<1%, Fig. 2A). Interestingly, NPCs grown as monolayers presented a well-known capacity to respond differentially to external cues. Thus, removal of FCS from the differentiation medium led to a significant increase of the number of βIII-tubulin positive cells (i.e. 5,7% ± 3,2, Fig. 2C). Finally, proliferative cells could be frozen and thawed with no change to their described properties (data not shown).

To confirm the presence of multipotent NPCs in our cultures, the capability of cells grown as monolayers to form neurospheres was assessed using clonal density cultures and the differentiative properties of these neurospheres were assessed. Cells at passage 4 were cultured in non-adherent conditions at a concentration of 1 cell/10 μl[2], and the number of neurospheres formed were counted (Fig. 2E). Numerous neurospheres rapidly formed (10% of the initial plated cells). Importantly, the majority of neurospheres were able to produce both neurons and astrocytes (Fig. 2F). Similar results were obtained from later passage monolayer cultures (data not shown). Since neurospheres have been shown to derive from single cells in these culture conditions[2], this demonstrates the maintenance of multipotent NPCs in our monolayer cultures. Furthermore, it demonstrates the ability of these cells to proliferate in different culture conditions.

### Nucleofection as a non-viral technique for single/multiple gene expression in neural precursor cells

**Figure 3:**
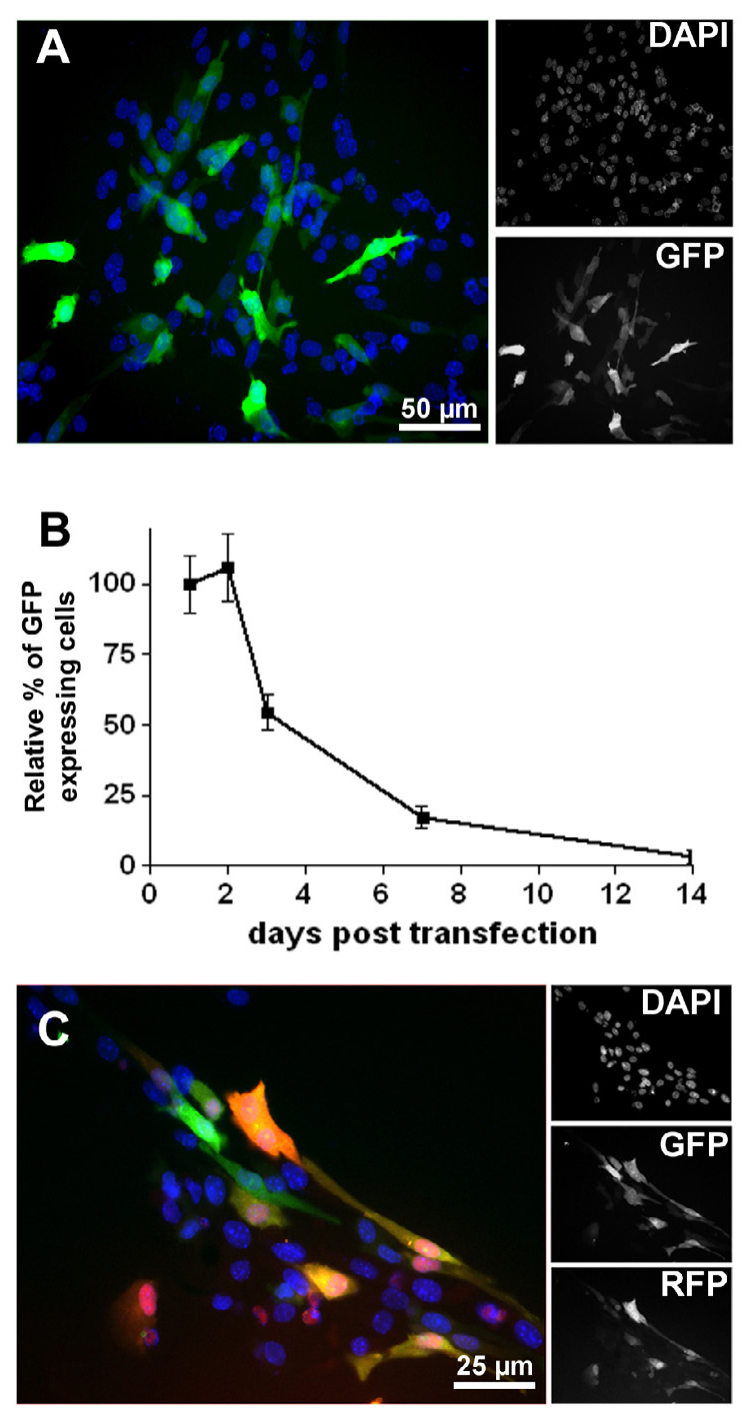
**Efficient nucleofection of single/multiple plasmids in mouse spinal cord NPCs**. **A**: Strong expression of the green fluorescent protein (GFP) coded by the control pMax plasmid (2 μg) was observed in ~30% of the cells at 48 h after transfection. **B**: Time course of GFP expression revealed a fast but transient expression of the reporter gene. **C**: Cotransfection with pMax (2 μg) and RFP (2 μg). Due to a weaker expression, RFP could only be detected in a subpopulation of GFP expressing cells. However, almost all RFP expressing cells were GFP positive (95% ± 3) demonstrating efficient and reliable plasmid co-transfection coul be achieved by nucleofection.

Nucleofection represents a rapid and efficient non-viral technique for nuclear gene delivery in numerous cell types. We first tested the transfection efficiency of our NPC monolayer cultures by using a plasmid (i.e. pMax plasmid, 2 μg) that coded for the green fluorescent protein (GFP). Forty-eight hours after transfection 30.2% ± 3.6 of the cells appeared intensely GFP positive (Fig. 3A). This rate of transfection was highly reproducible when identical transfection conditions (i.e. nucleofection program, cell number and plasmid concentration) were used. As expected, the expression of the transfected genes was very fast but transient in duration, with a peak of expression at 48 hours and a slow decrease thereafter to become almost undetectable at 14 days (Fig. 3B). The feasibility of concomitant transfection with multiple constructs was tested by co-transfecting cells with a GFP plasmid (2 μg) and a second plasmid (i.e. DsRed plasmid, 2 μg) coding for the red fluorescent protein (RFP). Virtually all the RFP-expressing cells (95% ± 3%, Fig. 3C) co-expressed GFP, demonstrating the potential of this technique for studying combinatory effects of genes on NPCs biology.

### Conditioned long-term YFP reporter gene expression

**Figure 4:**
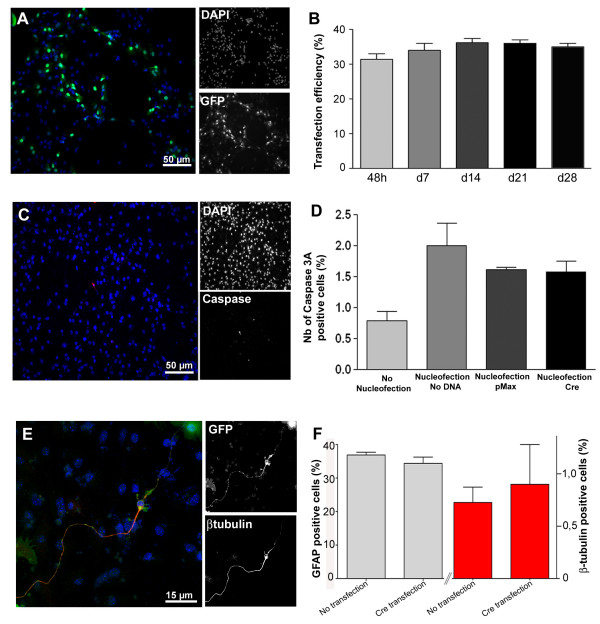
**Efficient and persistent YFP reporter gene expression induced by transient Cre expression in NPCs generated from Rosa26YFP transgenic mice**. **A-B**: Long-term YFP-expression was induced after transfection of NPCs with a Cre plasmid (2 μg). Transient expression of the Cre recombinase led to successful recombination in 30% of the cells. Note that this number remains stable over time, indicating an absence of down-regulation of the reporter gene. **C-F**: Cre-nucleofection was non-toxic and did not change the fate of mouse NPCs. Transient expression of Cre was not toxic for the cells as shown by immunodetection of the activated form of caspase 3, an apoptotic cell marker (Casp-3A, red, **C**). Quantification of the number of Casp-3A positive cells 48 h after nucleofection (**D**) showed an increase in cell apoptosis that was not exacerbated by Cre-expression. Note that >98% of the cells are Casp-3A negative 48 hours after nucleofection. Numerous cells co-expressed the reporter gene YFP and βIII-tubulin 48 hours after Cre transfection (**E**). Quantification of the number of GFAP and βIII-tubulin positive cells at 48 hours post cre-transfection showed no change in the fate of differentiated NPCs (**F**).

As mentioned above, nucleofection led to a fast but transient transgene expression, therefore preventing long-term tracking of the transfected cells. To circumvent this limitation, NPCs were isolated from cre-reporter mice (see above). We first tested the efficiency of YFP-reporter-gene-induction by transfecting Rosa26YFP-derived NPCs with a plasmid coding for the Cre-recombinase bacterial enzyme. Transient expression of Cre using nucleofection was sufficient to induce expression of the YFP-reporter gene as early as 48 h post transfection (Fig. 4A). The transfection efficiency was around 31,5% ± 2,7, similar to that observed with the GFP-plasmid (see above). Importantly, no YFP staining was observed in sister cultures that had not been transfected with the Cre plasmid. Critically, no down-regulation of the YFP expression was observed even after 28 days *in vitro*, as indicated by the stable number of cells expressing the reporter gene (Fig. 4B).

Because previous reports have indicated a potential cytotoxic effect of sustained Cre expression, we tested the potential toxicity of our transient transfection approach. Nucleofection of NPCs with a GFP plasmid or with a Cre plasmid was performed and the number of apoptotic cells was assessed by immunodetection of activated-Caspase 3 (Casp-3A, Fig. 4C). Nucleofection increased the number of Casp-3A positive cells at 48 hours (1,99% ± 0,73), when compared to non-transfected cultures (0,8% ± 0,25, Fig. 4D). Importantly, the number of apoptotic cells was comparable when cells were transfected with the control GFP plasmid or the Cre plasmid (1,6% ± 0,1 vs. 1,6% ± 0,5, respectively). Moreover, long-term culture of Cre-transfected cells showed a slight increase of the number of YFP expressing cells (from 31% at 48 h to 35% at 28 days, Fig. 4B) suggesting limited proliferation of a small population of the transfected cells, further supporting the conclusion of a non-toxic effect of the transient Cre expression.

Following nucleofection, cells were differentiated and their multipotentiality was assessed by immunostaining. No differences in the differentiation ability of Cre-transfected and non-transfected cells were observed (Fig. 4E). Indeed, the neuronal differentiation rate was 0,9% ± 0,53 for Cre-transfected cells (βIII-tubulin staining, Fig. 4F) versus 0,73% ± 0,35 for non-transfected cells. Astrocytic differentiation (i.e. GFAP positive cells), was 34,5% ± 3,3 and 37% ± 4,2 respectively for Cre-transfected and non-transfected cells. These data indicate that neither the fate of NPCs, nor the survival rate of neuronal and glial progenitors, is influenced by the nucleofection approach.

**Figure 5:**
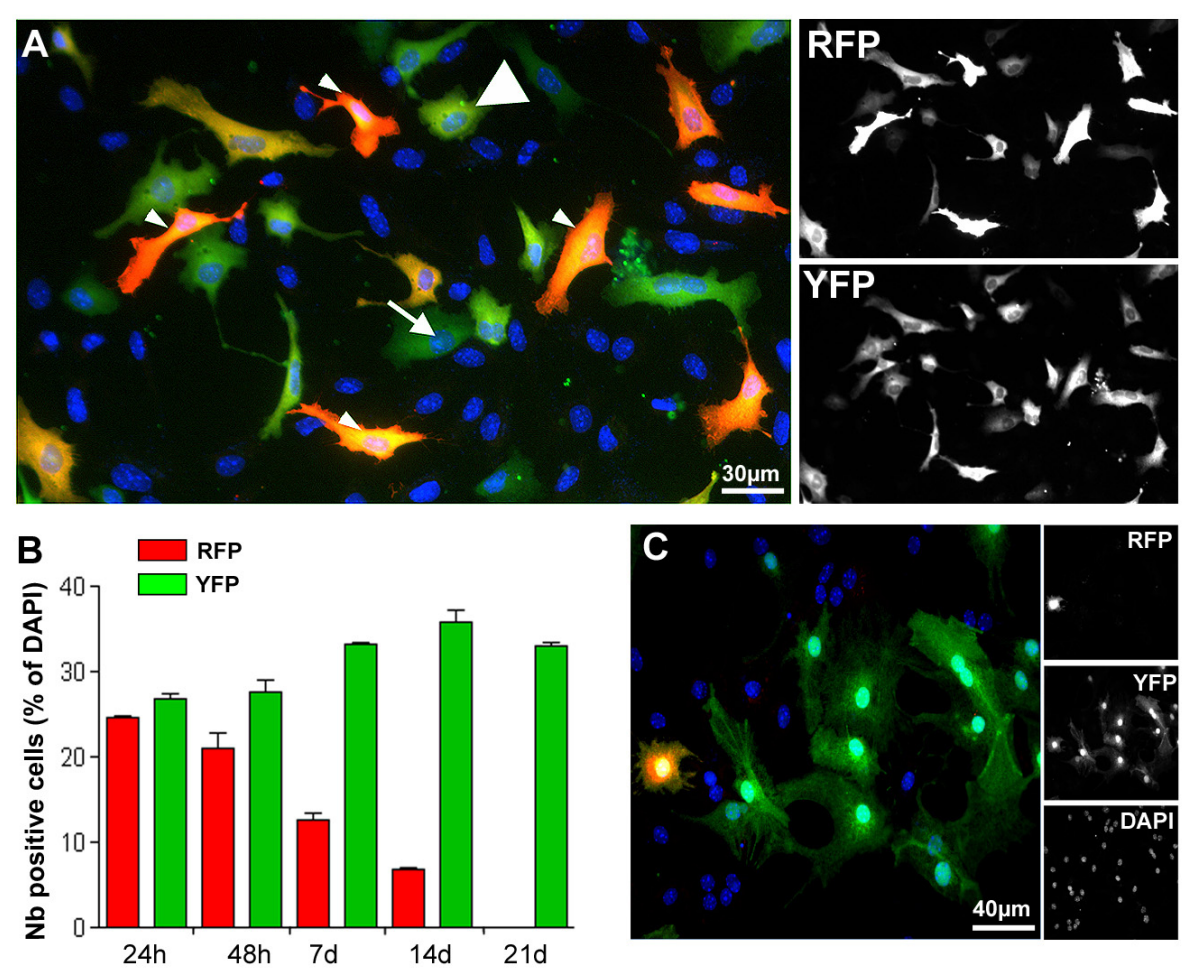
**Transfection of a bicistronic plasmid RFP-Cre led to transient expression of RFP and a stable expression of YFP**. **A**: Co-expression of RFP with the Cre-induced YFP reporter gene was observed in virtually all the cells at 24 h after nucleofection, demonstrating that Cre-induced YFP expression faithfully reflected the pattern of nucleofection. Note that the level of RFP expression was variable among the cells, but that recombination was efficiently induced in all cells. Small arrowheads present cells expressing high level of RFP whereas big arrowheads present cells expressing a low level of RFP. Arrow highlights an YFP positive cell negative for RFP. **B**: Quantification of the number of RFP and YFP-positive cells at different time point post-nucleofection demonstrated the transient expression of RFP but persistent, long-term expression of the YFP reporter gene. Note that the efficiency of recombination using a bicistronic plasmid was similar to that achieved with monocistronic Cre nucleofection. **C**: At 14-days-post-transfection, only few cells still expressed RFP whereas the YFP induced expression remained stable.

We next tested transfection with a bicistronic plasmid containing a reporter gene (i.e. RFP) followed by an internal ribosome entry site (IRES) and the Cre recombinase gene (i.e. DsRed-Cre plasmid). These plasmids allowed us to determine the exact time course of transgene expression and to test the efficiency of bicistronic Cre plasmids in the induction of recombination and, therefore, long-term reporter gene expression in the transfected cells. DsRed-Cre plasmid transfection led to rapid expression of both RFP and YFP in the transfected cells. Co-expression of the two reporter genes was observed in 92,5% ± 1,2 of the transfected cells at 24 h (Fig. 5A), indicating a rapid induction of the cre-reporter gene similar to that observed for the single gene transfection (see above). RFP immunodetection revealed various level of the protein expression in the transfected cells (Fig 5A). Interestingly, even low levels of expression lead to successful recombination and YFP expression (Fig 5A &5B). As previously observed for the control GFP plasmid, RFP expression was quickly down regulated from 24,5 ± 0,3 at 24 h to 21% ± 2 at 48 h (Fig. 5B &5C). Only 6,8% ± 0,3 of the transfected cells remained RFP-positive at 14 days, and no red cells could be detected at later time points. In contrast, cre-induced YFP reporter expression was 26,1% ± 0,1 24 h after transfection, and remained stable at ~30% for the entire culture period (i.e. 21 days, Fig. 5B), comparable to that observed for the Cre plasmid alone. Unlike for the RFP-expression, cre-induced YFP expression showed a similar level of expression within and among the cells (Fig. 5A &5B), as expected for a constitutively expressed reporter gene. These data indicate that the transient expression of genes can be reliably obtained through the use of a bicistronic plasmid. Furthermore, the induction of an ubiquitously and permanently expressed cre-reporter gene that faithfully reflects the pattern of transient transfection would facilitate the long-term fate tracking of such cells.

Taken together, these results demonstrate that transient Cre expression is sufficient for the induction of the expression of a permanent reporter gene in the transfected cells, with no toxic side effects.

### Long-term tracking of transplanted Cre-transfected neural precursor cells

Confirmation of our novel approach *in vivo *was performed by transplanting Cre-transfected NPCs into the brains of adult mice. Cultured NPCs were transfected with a cre-plasmid and were allowed to recover for 12 h before being transplanted into the adult corpus callosum and overlying cortex. To assess the fate and survival of the transfected cells, and the long-term induced-YFP-expression, animals were sacrificed 6 days and 1 month after transplantation.

**Figure 6:**
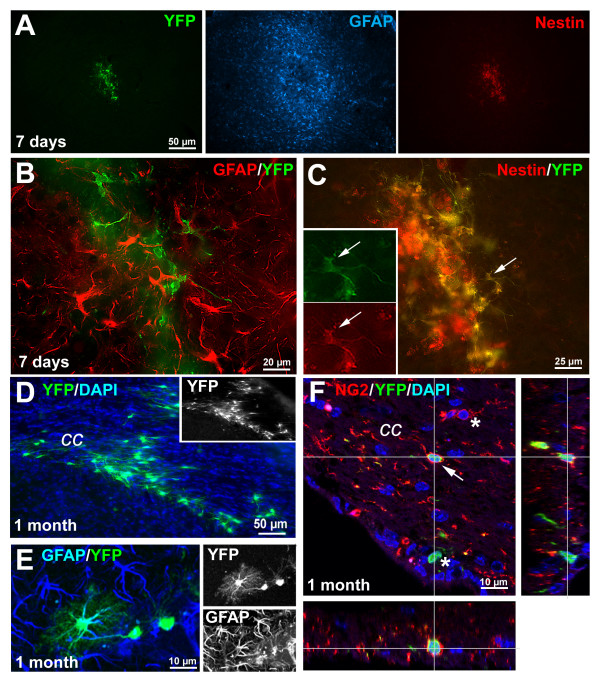
**Long-term YFP expression by Cre transfected NPCs *in vivo *after transplantation into adult mouse brains**. **A-C: **At 7-days-post-transplantation, cells did not migrate and remained as bolus at the injection site (**A**). Cells co-expressed YFP and Nestin (**C**) but not GFAP (**B**). Note the strong reaction of the tissue surrounding the graft as revealed by GFAP immunostaining at this early time point. **D-E**: At one month post-transplantation, intense migration of YFP-positive cells from the transplantation site along the corpus callosum was observed in all animals (**D**). **E**: Projection of a confocal stack spanning 15 μm in the z-dimension, showing a typical example of a fully differentiated YFP-positive cell expressing the astrocytic marker GFAP at 1 month post-transplantation. **F**: 3-dimentional projection of a confocal stack spanning 25 μm in the z-dimension, showing a typical example of a fully differentiated YFP-positive cell expressing the oligodendrocyte marker NG2 (arrow) in the corpus callosum at 1 month post-transplantation. Note the presence of NG2+/YFP- and YFP+/NG2- cells (asterisks) in the same region. ***cc***: corpus callosum

Seven days following transplantation, many YFP positive cells were observed at the injection site (Fig. 6A and Additional file 1A). Cells remained as a highly dense bolus at the injection site with minimal signs of migration. The cells presented a compact cell body and displayed a unipolar or bipolar morphology. This immature appearance correlated with the immunodetection of nestin in most of the cells (Fig. 6C). However, no cells expressed either neuronal (βIII-tubulin, NeuN) or glial (GFAP, Fig. 6B) markers. At 1 month post-transplantation, numerous YFP-expressing cells were still present (Fig. 6D and Additional file 1B). Evidence of extensive migration and differentiation was observed. Migration of transplanted cells from the injection site to the contralateral side of the brain, via the corpus callosum, was noted. These migrating cells were polarized, with small somas and long extensions typical of maturing oligodendrocytes. They were often positive for the oligodendrocyte marker NG2 (Fig. 6F). Other cells in the grey matter showed a more compact, multibranched morphology typical of astrocytes. Such cells expressed the marker GFAP, supporting their acquisition of an astroglial phenotype (Fig. 6E). In agreement with previous reports, no cells were seen to express the neuronal markers β III-tubulin or DCX (data not shown). Identical results were obtained when bicistronic Cre-containing plasmids were used to transfect the NPCs (data not shown).

These results suggest that the NPCs differentiate according to environment cues. More importantly, no evidence of dramatic reporter gene downregulation was observed suggesting that our Cre system can be used for fate determination and long-term tracking of nucleofected cells after transplantation.

## Discussion

Neural precursor cells (NPCs) exist in different regions of the CNS and can be isolated from both neonatal and adult mice[4]. The demonstration of their existence, together with their pluripotency and expansion capabilities, gave birth to the idea that they could be used for neuronal cell replacement therapies[7]. However, the fate of these cells is largely dependent on environmental factors. Thus, transplantation of these cells into the vast majority of the postnatal CNS leads to a predominant astrocytic differentiation[8,9]. The cascade of molecular events leading to fate decision in NPCs is beginning to be elucidated. Recent studies indicate that direct manipulation of key transcription factors represents a powerful strategy to manipulate the fate of these cells *in vitro*[12,14,19,20] as well as, albeit to a lesser extent, *in vivo *after transplantation[13]. Here, we have established a novel technique that allows transient single/multiple gene expression in mouse NPCs while also enabling long-term tracking of the transfected cell progeny fate.

To permit long-term tracking of the transfected cells, NPCs were isolated from postnatal Rosa26YFP cre-reporter mice[21,22]. In these mice, the insertion of the YFP reporter gene at an ubiquitously expressed locus allows conditional YFP expression in all cell types and visualisation of the entire transfected-cell progeny.

Isolation and long-term culture of postnatal mouse NPCs has mainly been performed using the neurosphere assay. While highly efficient, this preparation has some inherent limitations that restrict its use for transfection studies and cell fate analysis. Thus, the tightly packed cells of a neurosphere are difficult to transfect, while the analysis of their proliferation and differentiation is tedious. We have, therefore, developed a protocol to isolate and maintain postnatal CNS NPCs as monolayer cell cultures. This methodology is based on a previous study by Ray and Gage[4], with some modifications. Most notably, while Ray and Gage grew mouse NPCs directly onto uncoated flasks, we used an intermediate neurosphere formation approach. That is, immediately after a primary neurosphere formation, cells were dissociated and plated onto a polyornithine-laminin substrate. This difference in the adherent properties of the cells is unlikely to result from a difference in origin of the cells (brains for Ray and Gage vs. spinal cord in our case), as we have also successfully used the culture technique described here to isolate and culture NPCs from embryonic and neonatal hippocampi (data not shown). Initial isolation of NPCs using the neurospheres assay, therefore appears to have a major impact on the mouse cells adherent properties. Our NPCs, isolated from the cre-reporter mouse spinal cord grew as a monolayer and exhibited characteristics of precursor cells. Indeed, these cells expressed the undifferentiated neural cell markers nestin and Sox2, presented a proliferation rate stable over numerous passages, could reform neurospheres under clonal conditions, and were able to differentiate into neurons, astrocytes and oligodendrocytes upon removal of mitotic factors[23]. Consistent with the presence of multipotent stem cells in our monolayer cultures, was the generation of clonal neurospheres with the capacity to generate both neurons and astrocytes from the monolayer cultures. Furthermore, no drop in the proliferative properties of the cells was observed when early (P4) and late (P26) passaged monolayer cultures were compared.

Retro- and lentiviral techniques have previously been shown to transduce mouse NPCs efficiently[12,24]. However, the production of viruses is time consuming, requires special safety conditions and may present immunogenic, as well as safety concerns, for future clinical trials. Moreover, the use of viruses for gene manipulation presents several limitations, i) the sustained expression of transcription factors which may be toxic to the cells[15], ii) downregulation of reporter gene is often observed upon differentiation[13,25], iii) co-transfection of several genes is unreliable. This underlines the need for the development of non-viral techniques, which permit both the manipulation of single/multiple transcription factors in a rapid and efficient manner and the long-term tracking of manipulated cells to assess their fate and integration. In the present study, nucleofection (an electroporation based technology designed to introduce plasmid DNA directly into the cell nucleus) led to reproducible and efficient transfection of NPCs grown as monolayers. Non-viral transfection using nucleofection has been proved to be highly efficient using several cell types[26,27], including NPCs[28]. The transfection efficiency obtained in our study may appear low when compared to previous studies (30–35% vs. 60–80% [27,29]). This may be explained by differences in cell type, nucleofection programme used, nucleofector solution, quantity of DNA transfected as well as the promoter present in the plasmid transfected. Moreover, in our study the transfection rate were based on visual quantification of YFP and/or RFP expressing cells. This method of quantification is less sensitive than the fluorescent-activation cell sorting used in some of these previous studies. Critically, we could not detect any changes in the intrinsic properties of the cells following nucleofection, as suggested by others[29,30]. Moreover, this transfection technique allowed reliable cotransfection of NPCs, facilitating the study of the interaction between different genes/proteins. This is important as it is likely that several genes must be manipulated concomitantly within NPCs in order to generate cells of a given phenotype[31].

For long-term tracking of transfected cell progeny, a conditional cre-lox approach was developed. Using the nucleofection system, we were able to induce recombination of the loxP sites and, thus, trigger expression of the YFP cre-reporter protein to enable cell tracking. Sustained Cre expression has been shown to led to cell death, growth inhibition and DNA damage[32,33]. This, however, may be avoided by controlled expression of this protein[34,35]. Here, we report that transient Cre-expression is non-toxic, as addressed by immunodetection of the activated form of Caspase-3, and does not affect the proliferation rate and morphology of the transfected cells. In addition, we show evidences that transient Cre-expression does not perturb NPCs fate specification. This is in agreement with *in vivo *studies in which the cre-recombinase is expressed in adult hippocampal NPCs[36,37], with no reported side-effects. Transient transfection of the recombinase Cre using both mono- and bicistronic plasmids led to efficient and stable expression of YFP in Rosa26YFP cre-reporter mouse NPCs. No downregulation of the YFP reporter gene expression could be detected either *in vitro *or *in vivo*, up to 1 month post transfection. In order to exhibit the potential of our novel approach, cultured NPCs were transfected with a cre-plasmid. They were allowed to recover for 12 h before being transplanted into the adult corpus callosum and overlying cortex. Exploiting the long-term YFP reporter gene expression, cells were shown to survive transplantation and to gradually express mature glial morphology and specific markers. Thus, at 1 month post-engraftment cells had migrated extensively along the corpus callosum to the ipsi- and contralateral hemispheres in both the rostral and caudal directions, where they adopted complex glial morphologies. This robust migration along white matter tracts has been previously described in the intact brain[38] or after demyelination[39]. Their differentiation was demonstrated by the dramatic downregulation of nestin expression in the grafted cells at 1 month post-transplantation, and the concomitant upregulation of the glial markers GFAP and NG2. This time course and glial fate adoption of the transplanted cells in the adult brain is in agreement with previous reports[38]. Experiments using bicistronic plasmid gave similar results.

## Conclusion

In summary, we have developed a powerful new technique that allows the rapid, safe, and transient expression of single/multiple genes in NPCs cultured as monolayer, while simultaneously permitting the long-term tracking of the transfected cells and their progeny. This method will be valuable for testing the effect of single/multiple transgene manipulation on NPC fate determination both *in vitro *and *in vivo*. Furthermore, the use of cells isolated from specific cre-reporter mice, will allow the focused analysis of specific populations of cells, whenever needed. For example, NPCs isolated from Thy1-GFP[40] or Tau-GFP[41] cre-reporter mice can be used, resulting in the labelling of the neuronal progeny only, therefore greatly facilitating the quantification and analysis of this cell population. Alternatively, cells may be isolated from the Z/EG cre-reporter mice[42]. These cells express LacZ before recombination and GFP after, allowing direct comparison of the fate of transfected vs. non-transfected cells to be performed *in vitro*, or *in vivo *following transplantation.

## Materials and methods

### Cell culture

All experiments were done in agreement with the United Kingdom Animals Act 1986 for scientific procedures.

Neural precursor cells (NPCs) were isolated from the spinal cord (SC) of newborn postnatal day 5 (P5) Cre-reporter mice (Rosa26-YFP[21]) following a procedure modified from Ray and Gage[4]. Briefly, animals were killed by an overdose of pentobarbital (450 mg/kg, i.p.) and placed onto ice. The spinal cords were rapidly dissected and immediately immersed in ice-cold Hanks' balanced salt solution (HBSS, Ca2+/Mg2+ free, Invitrogen, UK). The meninges and blood vessels were carefully removed under observation with a binocular microscope. The spinal cords were sectioned into segments of ~1 mm^3 ^and incubated for 10 minutes in a solution containing 0.01% w/v papain (Worthington, UK), 0.1% w/v dispase II (Invitrogen, UK), 0.01% w/v DNAse I (Worthington, UK), 0.05 M L-cysteine-HCl and 12.4 mM MgSO_4_. The dissociated cell suspension was then filtered through a 40 μm cell sieve (Sigma, UK). Cells were centrifuged (1000 rpm, 5 min) and then washed three times in DMEM/F12 medium (Dulbecco's Modified Eagle Media, 1:1, Invitrogen, UK).

Cells were first cultured as neurospheres in an uncoated T25 flask in a culture medium consisting of Neurobasal A medium (NBA, Invitrogen, UK), 1% B27 (Invitrogen, UK), EGF (20 ng/ml, Sigma, UK), FGF2-Heparin (FGF2: 20 ng/ml, R & D system, UK, Heparin: 5 μg/ml, Sigma, UK), L-glutamine (2 mM, Invitrogen, UK) and 1% penicillin-streptomycin-fungizone mixture (PSF, Fisher, UK). Neurospheres usually formed after 7 days and were then passaged. Free-floating spheres were collected and washed three times in HBSS (Ca2+/Mg2+ free). Following trituration with a fired polished pipette, accutase (Sigma, UK) was added to the neurosphere suspension for 15 to 30 minutes at 37°C. Cells were triturated again with a fired polish pipette and the accutase was removed by washing the cells with a mix DMEM/F12 (1:1, Invitrogen, UK). To establish a monolayer culture, the single cell suspension was finally plated onto polyornithine/laminin-coated flasks (Sigma, polyornithine, PORN: 10 μg/ml, laminin: 5 μg/ml), in a medium consisting of DMEM/F12 (1:1), N2 supplement (1%, Invitrogen, UK), FGF2-heparine (as above), EGF (20 ng/ml) and PSF. The cells were plated at a concentration of 300,000 cells/ml and were fed every two/three days by replacing 50% of the medium with fresh medium (containing fresh EGF and FGF-2). When confluent, the cells were passaged using a brief treatment with accutase (30 sec to 1 min).

For differentiation, cells were plated onto PORN/laminin-coated coverslips at 200,000 cells/ml in a 24-well-plate (500 μl per well) in differentiation medium (i.e. NBA, 1% PSF, 1% B27, L-glutamine (2 mM), with or without 1% FCS). Cells were cultured up to 31 days, with half of the differentiation media replaced every 2 to 3 days.

For clonal studies, cells cultured as a monolayer (from passage 6 to passage 13) were grown as neurospheres at a clonal concentration (1 cell/10 μl [2]) in neurosphere medium (see above). Once neurospheres had formed, they were plated on PORN/laminin-coated coverslips in differentiation medium (see above) for 6 days.

To test the proliferative properties of the monolayer cultures, growth curves were realised by plating cells at a density of 1 × 10^6 ^per flask, and the total number of cells was counted daily from day 1 to day 4. Cultures at passage 6 and passage 26 were compared to assess proliferation after different periods *in vitro*.

### Plasmid production

Two plasmids containing the gene coding for the bacterial enzyme Cre-recombinase were used. The first plasmid contained the Cre-recombinase gene cDNA directly under the β-actin promoter (i.e. Cre plasmid). A second bicistronic plasmid, that contained the genes encoding the red fluorescent reporter protein DsRed, plus an IRES sequence followed by a nuclear localisation signal and the Cre-recombinase (i.e. DsRed-Cre plasmid), was constructed as follows. Firsts the gene DsRed2 was extracted by high fidelity PCR (pfu polymerase, Stratagen, UK) using the forward primer 5'aagcgcGGATCCGCCACCATGGCCTCCTCCG AGAACGTC3' (containing from 5' to 3' BamHI sequence, kozak sequence, N-terminus sequence of DsRed2) and the reverse 5'aagcgcGAGCTCCTACAGGAACAGGTGG TGGCG3' (i.e. SacI sequence, C-terminus sequence of DsRed2). The IRES-Cre-polyA fragment was extracted from the pCAGGS-Cre plasmid by digestion using SacI/XhoI. The two fragments DsRed2/Cre were finally ligated into a pCDNA3.1 (+) plasmid digested by BamHI/XhoI. The plasmid thus obtained was sequenced, analysed and amplified using the endotoxine free kit (Quiagen, UK).

### Nucleofection (amaxa)

Nucleofection of mouse spinal cord NPCs was performed using the mouse NSCs Nucleofector™ kit and optimised protocols provided by the manufacturer (Amaxa Biosystem, Cologne, Germany). Briefly, 3 to 4 × 10^6 ^cells were resuspended in 100 μl of the mouse NSC Nucleofector™ solution, which was prewarmed to room temperature. The cells were mixed with 2 μg of DNA, transferred into an amaxa certified cuvette and transfected with the program A-33. Immediately after transfection, 500 μl of the 37°C pre-warmed culture medium was added, and cells were plated onto PORN/laminin coated coverslips at a final concentration of 200,000 cells/ml (100,000 cells/well) in differentiation medium. Medium was changed three hours after plating (i.e. when all cells had adhered) to remove debris. Thereafter, cells were fed every second day by replacing 1/3 of the medium with fresh medium.

### Cell transplantation

After nucleofection of NPCs with a Cre-plasmid, the cells were plated onto PORN/laminin for 12 hours, in monolayer medium containing EGF/FGF-2 (see above). They were then gently resuspended at a concentration of 50,000 cells/μl in DMEM and stored on ice until transplanted. Cell viability (> 90% of the cells) was assessed at the end of each transplantation experiment by performing a trypan blue exclusion assay.

Adult mice (2 to 3 months old) of the same strain as the isolated cells (i.e. C57BL/6) were used to avoid immune rejection. The animals were anaesthetized by isoflurane inhalation and secured on a stereotaxic table. One microliter of the cell suspension was stereotaxically injected into the corpus callosum and overlying cortex of each animal (anteroposterior 2 mm; mediolateral 1,8 mm; dorsoventral 1,2 mm). Cells were slowly injected using a 5 μl Hamilton syringe over a 3 minute period, and the needle was maintained in place for 3 additional minutes before removal.

### Tissue processing

Animals were sacrificed at different time points following cell transplantation (i.e. 6 days, 1 month, 3 months) by overdose of pentobarbital (100 mg/g body weight, i.p.) and perfused transcardially with 10–30 ml of ringer solution followed by 30–50 ml of ice-cold paraformaldehyde (4% PFA) in phosphate buffer (PB 0.1 M, pH 7.4). Brains were removed from the animals, post-fixed overnight in the same fixative buffer and transferred to a solution of 30% sucrose in 0.1 M PB for cryoprotection. Forty micrometer sagittal sections were cut on a freezing microtome, collected in 0.1 M PB and processed as free floating sections for immunohistochemical characterization of the transplanted cells.

### Immunohisto- and immunocytochemistry

Similar primary antibodies were used for staining of the cell cultures and of the brain sections. The following concentrations were used: rabbit α-Caspase3 activated (1:1500, R & D systems, UK), mouse α-βIII-Tubulin (1:300, Sigma, UK), rabbit α-GFAP (1:400, Molecular Probes, UK), guinea-pig α-GFAP (1:400, Advanced ImmunoChemical, USA), mouse α-O4 (1:5, Developmental Studies Hybridoma Bank, USA), mouse α-Nestin (1:100, Developmental Studies Hybridoma Bank, USA), mouse α-DCX (1:500, Santa Cruz Biotechnology, USA), mouse α-NeuN (1:300, Chemicon, UK), rabbit α-GFP (1:300, Molecular Probes, UK), rabbit α-RFP (1:500, biotinylated, Abcam, UK), rabbit α-NG2 (1:500, Chemicon, UK).

#### Immunocytochemistry

Immunostaining against the cell surface oligodendrocyte marker O4 was performed on living cells. Differentiation medium was replaced by fresh medium containing mouse α-O4 antibody for 1 hour at 37°C. Cells were washed once with differentiation media and fixed with ice-cold 4% PFA in 0.1 M PB for 30 minutes. Immediately after fixation, the cells were washed 3 times 5 minutes in 0.1 M PB. Cells were incubated in PB-2%NHS with a secondary antibody α-mouse (Alexa-488 or Alexa-568, 1:1000, Molecular Probes, UK) for 2 h at 4°C, and then washed 3 times with PB. Blocking of unspecific antibody binding and membrane permeabilisation were achieved by incubating the cells for 2 hours at room temperature in 0.1 M PB, 0.4% Triton ×100 containing 10% normal horse serum (PB-Tx-10%NHS). Cells were washed three times with 0.1 M PB-Tx, and were incubated with primary antibodies in PB-Tx-2%NHS for 2 h (GFP, RFP, GFAP) or overnight (Nestin, βIII-tubulin, DCX) at 4°C on a shaker. Following three washes, cells were incubated in PB-Tx-2%NHS for 2 hours at 4°C with secondary antibodies α-mouse, α-goat, α-rabbit or α-guinea pig (Alexa-488, Alexa-546, Alexa-568, Alexa-660, 1:1000, Molecular Probes, UK). For detection of the rabbit α-RFP biotinylated antibody, cells were incubated for 10 minutes with streptavidin-Cy3 complex (Jackson Immunoresearch, USA) in PB-Tx. Secondary antibodies were washed out and a nuclear counterstaining was realised using 4',6-diamidino-2-phenylindole (0,1 μg/ml, DAPI, Sigma) for 5 minutes at room temperature. Following two additional washes in PB-Tx, cells were washed once in PB and mounted in Vectashield fluorescent mounting medium (Vector, UK).

#### Immunohistochemistry

Free-floating brain sections were blocked/permeabilized overnight at 4°C in PB-Tx-10%NHS. The sections were washed three times with 0.1 M PB-Tx, and then incubated with primary antibodies in PB-Tx-2%NHS overnight (all antibodies) or for 2 days (GFAP only) at 4°C. Following three washes, the sections were incubated in PB-Tx-2%NHS for 2 hours at 4°C with secondary α-mouse, α-goat, α-rabbit or α-guinea pig antibodies (Alexa-488, Alexa-546, Alexa-568, Alexa-660, 1:1000, Molecular Probes, UK). For optimal visualization of the GFP-positive cells, amplification of the GFP staining was realised using a biotinylated secondary goat anti-rabbit or donkey anti-rabbit antibody (1:1000, Jackson Immunoresearch, USA) followed by a 2 hours incubation with streptavidin-DTAF (1:500, Jackson Immunoresearch, USA) at 4°C. After several washes in 0.1 M PB, a nuclear counterstain was performed using DAPI (Sigma, UK) for 5 minutes at room temperature. Following two additional washes in PB-Tx, cells were washed once in PB and mounted in Vectashield fluorescent mounting medium.

### Fluorescent imaging and quantification

For acquisition of wide field fluorescent images, a Leica DM6000 microscope equipped with a Leica FX350 camera and the FW4000 software was used. At least 5 pictures from each slide were taken using a Leica ×20 objective (N.A. 0.7). Images of the different fluorochromes were captured using appropriate filter sets (i.e. cubes A5, L5, Y3, Y5, Y7, Leica Microsystems GmbH, Germany). The quantification was performed using Photoshop 7 image software (Adobe software incorporated, USA). For analysis of grafted cells, blind 3D deconvolution was performed using the Deblur extension of the FW4000 software (AutoQuant).

Confocal pictures were acquired on a Leica LCS-SPE confocal microscope using a Leica ×40 or ×63 objective (N.A. 1.2 and 1.4, respectively) and processed using the LAS-SPE software (Leica Microsystems GmbH, Germany).

Positive cells were quantified using at least five randomly chosen, non-overlapping fields for each coverslip. An average was calculated from at least three coverslips for each experiment. Data were obtained from triplicates (unless otherwise stated in the text). The data are expressed as mean values ± standard error of mean (SEM). Statistical analysis (student t-test) was carried out using Prism Software. Significant differences were assumed at a level of *P *< 0.05.

## Authors' contributions

CG carried out the experimental work. OR conceived and supervised the study. Both authors read and approved the final manuscript.

## Supplementary Material

Additional File 1 Low magnification of YFP expression by Cre transfected NPCs after transplantation into adult mouse brains. Low magnification (10×) photomicrographs showing the location of the YFP-expressing NPCs at 7-days-post-transplantation (**A**) and the intense migration of the cells through the corpus callosum at 1-month-post-transplantation (**B**). cc: corpus callosum; v: lateral ventricle. Click here for file
