# Supplementary figures and images for: A Cre-lox approach for transient transgene expression in neural precursor cells and long-term tracking of their progeny in vitro and in vivo
Source: BMC Dev Biol. 2007 May 15;7:45. doi: 10.1186/1471-213X-7-45 (PMC1885435; doi:10.1186/1471-213X-7-45)

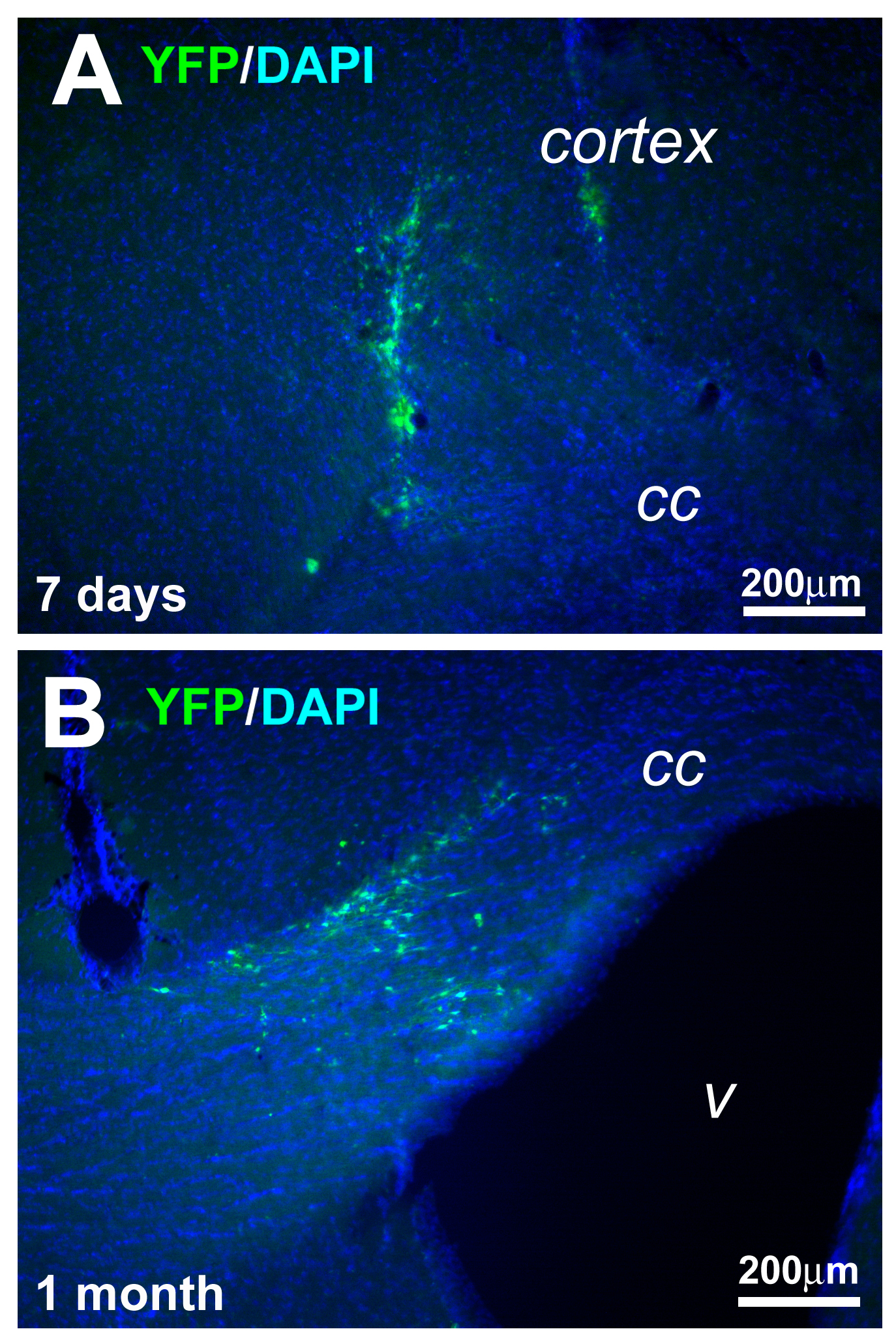

Supplement: Additional File 1 — Low magnification of YFP expression by Cre transfected NPCs after transplantation into adult mouse brains. Low magnification (10×) photomicrographs showing the location of the YFP-expressing NPCs at 7-days-post-transplantation (A) and the intense migration of the cells through the corpus callosum at 1-month-post-transplantation (B). cc: corpus callosum; v: lateral ventricle. [file 1471-213X-7-45-S1.jpeg]
